# A comparative hidden Markov model analysis pipeline identifies proteins characteristic of cereal-infecting fungi

**DOI:** 10.1186/1471-2164-14-807

**Published:** 2013-11-20

**Authors:** Jana Sperschneider, Donald M Gardiner, Jennifer M Taylor, James K Hane, Karam B Singh, John M Manners

**Affiliations:** 1 Commonwealth Scientific and Industrial Research Organization (CSIRO) Plant Industry, Centre for Environment and Life Sciences, Perth, Western Australia, Australia; 2 CSIRO Plant Industry, Queensland Bioscience Precinct, Brisbane, Queensland, Australia; 3 CSIRO Plant Industry, Black Mountain Laboratories, Canberra, Australia; 4 The UWA Institute of Agriculture, University of Western Australia, Crawley, Australia

**Keywords:** Fungal pathogens, Comparative genomics, Effectors, *Fusarium graminearum*, Cereal host, Hidden Markov model, Protein structure

## Abstract

**Background:**

Fungal pathogens cause devastating losses in economically important cereal crops by utilising pathogen proteins to infect host plants. Secreted pathogen proteins are referred to as effectors and have thus far been identified by selecting small, cysteine-rich peptides from the secretome despite increasing evidence that not all effectors share these attributes.

**Results:**

We take advantage of the availability of sequenced fungal genomes and present an unbiased method for finding putative pathogen proteins and secreted effectors in a query genome via comparative hidden Markov model analyses followed by unsupervised protein clustering. Our method returns experimentally validated fungal effectors in *Stagonospora nodorum* and *Fusarium oxysporum* as well as the N-terminal Y/F/WxC-motif from the barley powdery mildew pathogen. Application to the cereal pathogen *Fusarium graminearum* reveals a secreted phosphorylcholine phosphatase that is characteristic of hemibiotrophic and necrotrophic cereal pathogens and shares an ancient selection process with bacterial plant pathogens. Three *F. graminearum* protein clusters are found with an enriched secretion signal. One of these putative effector clusters contains proteins that share a [SG]-P-C-[KR]-P sequence motif in the N-terminal and show features not commonly associated with fungal effectors. This motif is conserved in secreted pathogenic *Fusarium* proteins and a prime candidate for functional testing.

**Conclusions:**

Our pipeline has successfully uncovered conservation patterns, putative effectors and motifs of fungal pathogens that would have been overlooked by existing approaches that identify effectors as small, secreted, cysteine-rich peptides. It can be applied to any pathogenic proteome data, such as microbial pathogen data of plants and other organisms.

## Background

Fungal pathogens represent one of the most important restraints of food production worldwide and can also threaten natural plant populations [1,2]. Any one plant species can come under attack from a diverse range of fungal pathogens that may not be related. These different pathogens may also have different modes of infection: biotrophs which require living tissue to obtain nutrients, necrotrophs which kill the plant cells to obtain nutrition and hemibiotrophs which show phasic interactions with plant hosts that are a combination of both biotrophy and necrotrophy. The outcome of the interaction between any of these infection modes will depend on both the ability of the plant to defend itself and the ability of the pathogen to cause disease. Understanding how diverse pathogens cause disease on specific hosts will provide information for the development of new crop protection strategies.

Pathogens utilise a wide range of virulence strategies to bring about disease. These include the production of small secreted proteins (or effectors) that interfere with host functions, the production of small secondary metabolites that may have effector-like function or be toxic to host cells and the production of reactive oxygen species. Pathogens also possess mechanisms to defend themselves against the host response to infection such as proteins to mask other molecules that may otherwise be recognised by the host, enzymes to metabolise toxic plant derived compounds and enzymes to detoxify reactive oxygen species. However, our understanding of the virulence arsenal of most pathogens is fragmentary. This is in part because most virulence strategies possessed by a single pathogen contribute in a quantitative manner to the outcome of an interaction, making their detection difficult. Proteinaceous effectors that act as avirulence determinants in specific host genotypes are an exception to a quantitative contribution to an interaction, but the virulence function of these in compatible hosts are also poorly defined.

*De novo* prediction of pathogen virulence mechanisms in fungi based on common sequence features has to date been difficult. In contrast, proteinaceous effectors of bacteria and oomycete pathogens can be predicted from conserved targeting signals in the N-terminal regions with comparative ease. Using whole-genome sequence and deduced protein-coding gene sequences of these organisms, proteins with certain conserved amino-acid motifs in their N-terminal region have been observed to be strongly associated with predicted secreted proteins. In bacteria, this is the signal that targets proteins to the type III secretion system which directly delivers proteins to the host cytoplasm [3-5]. Effectors in oomycete secretomes have conserved RXLR-dEER [6], Crinkler [7], CHXC [8] and YXSL[RK] motifs [9] allowing the identification of new Oomycete effectors. The RXLR-dEER motif has been demonstrated to facilitate effector uptake by the host cell [10-12]. oomycete genomes encode hundreds of such potential effectors [13], a growing number of which have been functionally validated.

In the fungi, no functional uptake signal has been described yet and thus robust effector prediction based on signature sequence motifs are not feasible. Effector detection in fungal pathogens has been based largely on host specific responses mediated by host recognition of the effectors [14]. Bioinformatics approaches utilize known effector features for analyzing the secretome of fungal pathogens to find proteins with possible roles in virulence. The secretome is typically predicted using a combination of signal peptide prediction methods [15,16] and accuracy relies heavily on the reliability of the secretion prediction methods used. Effector prediction in the literature commonly involves selecting small cysteine-rich proteins from the predicted secretome as these have been associated with properties of known effectors [15,17,18]. Small, cysteine-rich proteins are typically defined as being shorter than 150 amino acids [19] and having more than four cysteine residues [20], however there is no consensus in the literature about how these thresholds are set. Therefore, this type of selective effector prediction approach is dependent on the validity of the thresholds used and also makes *a priori* assumptions about effector protein properties.

These a *priori* assumptions about effectors are beginning to be challenged with the discovery of effector proteins that are larger in size, do not contain a high number of cysteines and are not predicted to be secreted based on a signal peptide sequence. For example, a 290 amino acid secreted chorismate mutase enzyme of the Basidiomycete fungus *Ustilago maydis* is taken up by maize cells where it interferes with the production of the defence-stimulating plant hormone, salicylic acid [21]. In the bacterium *Xanthomonas campestris* a 536 amino acid uridine monophosphate transferase acts as a virulence effector inibiting host kinases [22]. The *SIX3* gene in *Fusarium oxysporum* encodes an effector protein with only two cysteine residues [23] and the *AvrM* gene encodes an effector without cysteines in the haustoria-forming pathogen *Melampsora lini*[24]. The barley powdery mildew fungus genes *AVR-a10* and *AVRk1* encode effector proteins without a secretion signal peptide [25]. Furthermore, in a proteomic study of the interaction between *F. graminearum* and wheat, a large proportion of proteins of fungal origin isolated from the apoplastic space between host plant cells were also not predicted to be secreted [26]. The reverse approach of functionally analysing large sets of secreted proteins for roles in virulence has to date also had a relatively low success rates. For example, in an analysis of 78 secreted proteins from *Magnaporthe oryzae* only one was shown to have a role in virulence [27]. In summary, we propose that fungal pathogens encode many more effectors than those currently known and with additional or alternate molecular properties than those that are predicted by current approaches.

An unbiased and powerful way to predict the virulence arsenal of fungal pathogens is to compare and contrast fungal pathogens via comparative genomic analyses. Fungal pathogens of the same or similar host plant can be expected to have evolved, to some degree, common virulence mechanisms that target the same host components to bring about disease. The relatively small size of fungal genomes (compared to many plant hosts) makes them amenable to whole genome sequencing and the number of fungal genome sequences available provides an unprecedented opportunity to use comparative analyses to associate genes with virulence functions. Cross species comparative analysis has been successfully applied to the identification of completely novel virulence mechanisms in wilt pathogens of dicot hosts [28] and in the cereal-infecting fungus *F. pseudograminearum*[29]. In both of these examples, proteins uniquely present in organisms sharing a common biological feature (i.e wilt-causing fungi or cereal-infecting fungi, respectively) were shown to be involved in virulence. Both of these studies relied on the sequence similarity search tool BLAST [30]. BLAST is a position-dependent local alignment tool that works well for regions of high sequence similarity. However, more sensitive methods are likely to be required in fast-evolving genome regions where mutations might lead to little sequence identity amongst a protein family, yet the three-dimensional structure, i.e. the functionality, will be preserved. For example, RXLR effector proteins might share as little as 15% sequence identity yet they can have almost identical three-dimensional structures [31]. Sequence similarity search tools such as BLAST will fail to detect those important functional relationships.

More sensitive interrogation techniques for screening for putative virulence and effector molecules in fungal pathogens are hidden Markov models (HMMs). Profile HMMs are an alternative to BLAST and are statistical models of multiple sequence alignments with the ability to capture position-specific information. Certain positions can have specific scores for amino acids as well as specific gap penalties for insertions or deletions. This is a clear mathematical and biological advantage over position-independent local alignment tools such as BLAST. Profile HMMs can group sequences into evolutionarily related families and find remote homologues. The power of profile HMMs becomes obvious when a family of evolutionarily related sequences is known and a carefully curated multiple sequence alignment is available. Instead of scanning a large number of weak BLAST hits by eye for remote homologues, a profile HMM can be calculated from the alignment and can be used to search a database for remote family members. Profile HMM searches are now essentially as fast as BLAST and available in the HMMER3 software package [32]. If no initial multiple sequence alignment is available, a profile HMM can be built from a single sequence and can be used in a database search using phmmer. Despite these advantages, to our knowledge, single sequence profile HMM methods such as phmmer have not been applied to the *de novo* identification of putative virulence-related proteins and effectors in pathogens.

**Figure 1:**
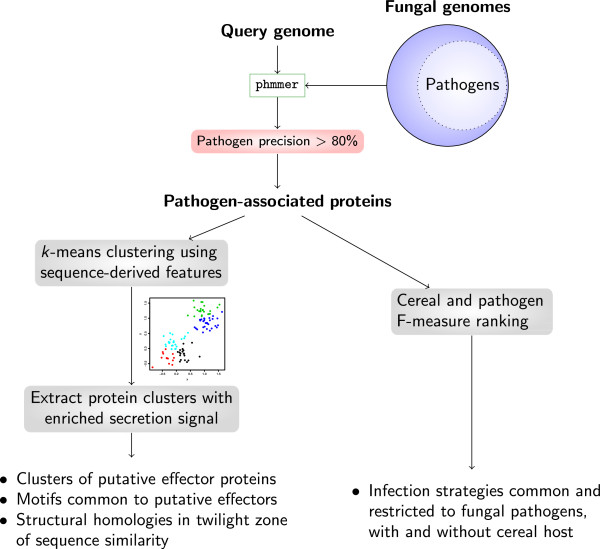
**The comparative pipeline for predicting candidate virulence genes and effectors.** Our comparative analysis pipeline predicts proteins in a query pathogen genome which have HMM sequence similarity hits predominantly in fungal pathogens. For a protein to be associated with pathogenicity, the list of phmmer hits must include at least 80% hits to proteins from pathogen species (including the query genome itself). These pathogen-associated proteins are then analyzed in terms of two criteria: (1) their degree of conservation across other fungal pathogens with and without a cereal host and (2) their potential to act as effectors outside the fungal cell. Proteins which are highly conserved across a diverse range of other pathogens as identified by our *F*-measure ranking are prime candidates for virulence-related proteins. To identify putative effector candidates in an unbiased way, an unsupervised clustering technique based on 35 sequence-derived protein features is used to look for protein clusters with an enriched secretion signal. Further investigation of the clusters with regards to predicted sequence motifs, novel secretion signals, amino acid composition and functional annotation is conducted to find and characterise novel putative effector families.

In this work, we present a sensitive and accurate HMM-based pipeline for predicting proteins with shared functions, in this particular study with putative virulence or effector functions, based on a comparative analysis and a subsequent protein clustering step (Figure 1)*.* We use 174 fungal genomes from the Joint Genome Institute (JGI) MycoCosm [33], which covers diverse pathogenic and non-pathogenic fungi and is a well-established resource in fungal genomics [34]. Instead of restricting ourselves to a small number of genomes from a particular lineage (and thus creating taxonomic bias), we intentionally used 174 publicly available fungal genomes which cover the fungal kingdom in a phylogenetically and phenotypically diverse manner. The initial step of the pipeline is to find proteins in a query fungal pathogen genome which have HMM sequence similarity hits specific to fungal pathogens and at the same time predominantly absent from non-pathogenic fungi. These pathogen-associated proteins are ranked according to their degree of conservation across other fungal pathogens, with and without a cereal host, to find infection mechanisms common to pathogenic fungi. Unsupervised clustering of the pathogen-associated protein set according to 35 sequence-derived features (e.g. signal peptide prediction score, molecular weight, amino acid composition) leads to protein clusters which share similar characteristics. From this we identify clusters that have an enriched secretion signal or conserved potential signalling domain as putative effector protein groups. The major advantage of the unsupervised clustering of proteins for finding effector genes is that no heuristic cut-offs such as protein length or the number of cysteines is used. However, if these are features common to a group of effectors, they will show up in the clusters naturally. Furthermore, the clustering of pathogen-associated proteins using sequence-derived features is a first step towards finding structural homologies in the twilight zone of sequence similarity (20–30% pairwise sequence identity). Our method has revealed promising novel candidate virulence factors as well as putative effector molecules, some of which appear to have unusual evolutionary origins suggesting they have been subjected to specific selection and have important functions in pathogenesis. Furthermore, we have identified protein groups with related sequence-derived features, which may indicate common functionality. Our method can have wide application in effector discovery in microbial pathogens of plants and other organisms.

## Results and discussion

### Development of a candidate virulence gene selection pipeline

To identify genes with potential roles in virulence on plant hosts a prediction pipeline was developed that utilises distribution across the fungal kingdom as a means of identifying candidates. The hypothesis underlying this pipeline is that genes involved in virulence processes are more likely to be retained or uniquely present in organisms that share a biological trait such as pathogenicity towards plants. The comparative analysis pipeline queries a genome of interest by a systematic and sensitive HMM comparison to a large number of publicly available fungal genomes. Here, 174 genome sequences were used, but as new genomes are published they can be added to the pipeline. Our approach intentionally avoids taxonomic groupings of the 174 fungal genomes as pathogens can be phylogenetically closely related yet at the same time be phenotypically diverse, e.g. by infecting different hosts or tissues. For example, the group species *Fusarium oxysporum* contains plant and opportunistic animal pathogens, host-specific pathogens, non-pathogens and biocontrol agents.

**Figure 2:**
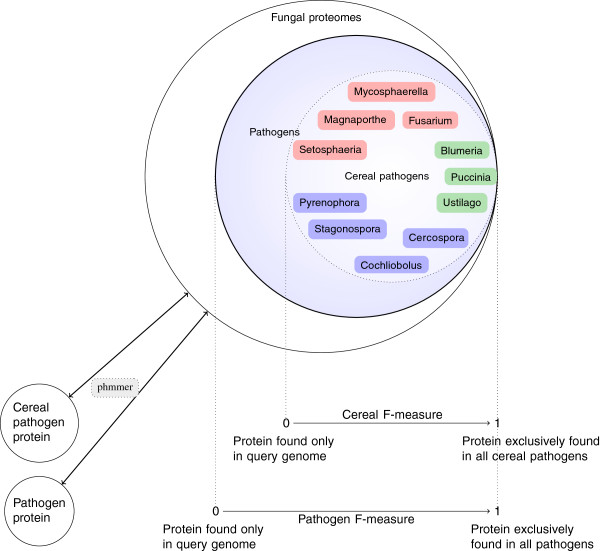
**A visualization of the *F*-measure for finding highly conserved virulence genes in pathogens, with and without a cereal host.** The set of 174 fungal proteomes from the JGI MycoCosm tree [33] is shown with the subsets of pathogenic fungi and cereal-infecting fungi. The genera of cereal-infecting fungi are colored according to fungal lifestyle (biotrophs, hemibiotrophs, necrotrophs). The *F*-measure is the harmonic mean of precision and recall and ranges from 0 to 1. For a query pathogen protein of interest, a *F*-measure of 0 means that the protein is only found in the query pathogen genome. A perfect pathogen *F*-measure of 1 means that this protein is found in all pathogen genomes and at the same time only in pathogenic fungi, thus indicating a protein highly relevant to pathogenicity. In the middle of the scale sit pathogen proteins which can be found mostly in pathogens and at the same time in some non-pathogens. We also introduce the more specific cereal *F*-measure to identify proteins which are highly conserved and exclusive to cereal-infecting fungi to identify host-specific infection patterns.

The initial step identifies proteins predicted in the query genome that are enriched in pathogen genomes and are predominantly absent in the genomes of non-pathogens (Figure 1). Thus, this step identifies putative virulence- and pathogenicity-associated proteins. The next step in the pipeline is to rank these pathogen-associated proteins according to their respective frequency of association across pathogens, and this was achieved using the *F-*measure, which is the harmonic mean of precision and recall. A perfect *F*-measure of 1 indicates presence uniquely in fungal pathogens and across all fungal pathogens (Figure 2). To investigate host-specific patterns, we report the *F*-measure with respect to two backgrounds, the frequency of association across pathogens in general (pathogen *F*-measure) and the frequency of association across pathogens with a cereal host (cereal *F*-measure). To dissect the nature of the pathogen-associated proteins we next used an unsupervised clustering method based on 35 features (e.g. signal peptide prediction score, molecular weight, amino acid composition), deduced from the sequences. This leads to a suite of clusters of related pathogen-associated proteins. One of our aims was to identify potential effector proteins, which we define as proteins active outside the fungal cell that interact with host defences as well as pathogen recognition and signalling by the host, and these would be expected to represent a subset of the pathogen-associated protein clusters. Traditionally, effector candidates have been identified as small secreted peptides, whereas our model makes no assumption on size, and also includes searches for novel potential secretion signatures, as well as conventional secretion signals. If a family of effectors has sequence-derived features in common, such as the amino acid composition or the predicted signal peptide score, and if these features are distinct from the remaining proteins, it can be expected to form a natural group or cluster. This cluster will then have an enrichment or depletion in its characteristic features from background, which is the set of all pathogen-associated proteins. At this stage, proteins for specific functional and evolutionary analysis can be identified to further elucidate roles in pathogenesis.

### Application to *S. nodorum* and *F. oxysporum* returns known effectors

*F. graminearum* is one of the most important pathogens of cereals globally and was the focus for our study. However, no effectors have been functionally validated so far in the literature for this pathogen. Therefore, we initially tested our pipeline on two fungal genomes which have a number of characterized effector proteins, i.e*. Stagonospora nodorum* SN15 [35] and *Fusarium oxysporum* f. sp. *lycopersici strain* 4287 [36]. Note that unlike many bacterial pathogens, no individual fungal pathogen has sufficient numbers of known virulence and non-virulence proteins to allow benchmarking by identifying true positives and false positive rates, which prohibits a large-scale validation of the pipeline.

*S. nodorum* SN15 is a necrotrophic pathogen of wheat with a number of characterized effectors or host-selective toxins, called SnToxA, SnTox1 and SnTox3. We applied our gene-selection pipeline to the predicted protein set of the *S. nodorum* genome and found 3528 proteins (28% of gene models) which are enriched in pathogen genomes and are predominantly absent in the genomes of non-pathogens (Additional file 1). The proteins with the highest combined degree of conservation and exclusivity across fungal pathogens according to the pathogen *F*-measure are all predicted to be secreted (SNOG_12346, SNOG_01958, SNOG_09549, SNOG_13500, SNOG_05633). Two of the proteins have a functional annotation, one as a putative secreted phosphorylcholine phosphatase (SNOG_09549) and another one as a predicted cystatin-like fold (SNOG_13500). The *S. nodorum* proteins with the highest cereal *F*-measure are a zinc finger domain containing protein (SNOG_09637), two small secreted cysteine-rich proteins with no functional annotation (SNOG_20011, SNOG_05982) and a putative secreted phosphorylcholine phosphatase (SNOG_09549).

**Figure 3:**
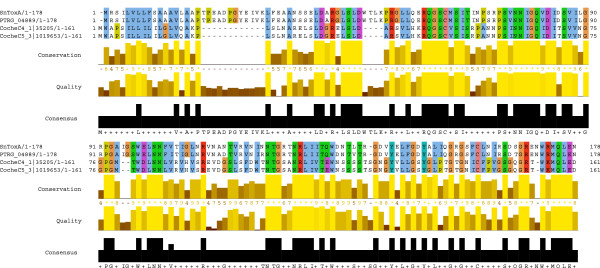
**HMM search returns ToxA-like proteins in *Cochliobolus heterostrophus *isolates C4 and C5.** A phmmer search with SnToxA (SNOG_16571) returns sequence similarity hits to *Pyrenophora tritici-repentis* (PTRG_04889) and hits to potential ToxA-like domains in two proteins in *Cochliobolus heterostrophus* isolates C4 and C5. The corresponding multiple alignment of the phmmer domain hits is visualized by Jalview [39].

Our predicted set of pathogen-associated *S. nodorum* proteins includes the known effectors SnToxA (SNOG_16571), SnTox1 (SNOG_20078) and SnTox3 (SNOG_08981). Overall, SnToxA was ranked 65^th^ in the cereal *F*-measure list. Due to some proteins sharing the same *F*-measure, the top 65 rank positions account for 636 of the 3528 proteins enriched in pathogen genomes. For the three known effectors SnToxA, SnTox1 and SnTox3, SnToxA has the highest cereal *F*-measure of 0.35 due to HMM sequence similarity hits to *Pyrenophora tritici-repentis* and *Cochliobolus heterostrophus* isolates C4 and C5 [37]. For both *C. heterostrophus* isolates C4 and C5 proteins a ToxA domain is predicted using the 3D structure prediction software Phyre2 (100% confidence, 44% sequence identity). Interestingly, despite the high level of sequence similarity, the RGD-containing loop [38] is not conserved in *C. heterostrophus* isolates C4 and C5 (SGN-containing loop, Figure 3). The other two host-selective toxins SnTox1 and SnTox3 are only found in *S.nodorum* and thus have the lowest possible *F-*measure. In general, the unsupervised clustering of pathogen-associated proteins returns three clusters with a significantly higher secretion signal than the background distribution for all clusters (Mann–Whitney *U* test, p-value < 2.2e-16). Interestingly, the three effectors SnToxA, SnTox1 and SnTox3 all belong to the same cluster, which has 191 members in total and is enriched in small and non-polar amino acids (data not shown). The 191 proteins in this cluster share sequence-derived similarites and based on the membership of known effectors there is good potential that this cluster includes proteins that are worth investigating in terms of their effector potential.

*F. oxysporum* f. sp. *lycopersici strain* 4287 is a hemibiotroph pathogen that causes vascular wilt in tomato plants. Our pipeline returns 4613 proteins (26% of gene models) which are enriched in pathogen genomes and are predominantly absent in the genomes of non-pathogens (Additional file 2). As *F. oxysporum* is not a cereal-infecting pathogen, we investigated the proteins with highest pathogen *F*-measure. These include a putative secreted phosphorylcholine phosphatase (FOXG_09820), a putative secreted peptidase C69 domain protein (FOXG_05778) and a putative synaptotagmin-1 protein fold (FOXG_13700). *F. oxysporum* is known to secrete several proteins during infection of the host and these are referred to as secreted-in-xylem (SIX) proteins, which include several functionally proven effectors [23,40-42]. Our set of predicted pathogen-associated proteins contains all the SIX proteins for this strain, i.e. SIX1 (FOXG_16418), SIX2 (FOXG_16416), SIX3 (FOXG_16398), SIX6 (FOXG_14246), SIX8 (FOXG_16464, FOXG_17445), SIX9 (FOXG_14223) and SIX10 (FOXG_17457). These effectors have lowest possible *F-*measure due to the lack of hits to any other fungal pathogen. The unsupervised clustering returns three clusters with a significantly higher secretion signal than the background distribution for all clusters (data not shown). For the SIX2 effector, the software SIGNALP 4.0 does not predict a signal peptide, yet in our analysis it is still placed in the same cluster as the secreted SIX1, SIX6, SIX9 and SIX10 effectors based on its sequence-derived features. This might indicate that our clustering approach successfully recognizes structural similarities amongst the pathogen-associated proteins. This particular cluster has 221 members and is enriched in tiny, small and non-polar amino acids and in particular in the amino acids cysteine, glycine and asparagine. Due to the occurence of known effectors in this cluster and the traditional association of some of the cluster characteristics (small, secreted, cysteine-rich) with effectors, this cluster might contain other potential effector candidates. The SIX3 and SIX8 proteins are also not predicted to be secreted by SIGNALP 4.0. SIX3 sits in a cluster with high average positive protein charge and a significantly higher percentage of prolines, serines and threonines than the other clusters. SIX8 is part of a cluster with a strong secretion signal and a significantly higher percentage of aliphatic amino acids than the background distribution for all clusters.

Taken together, the blind study of *S. nodorum* and *F. oxysporum* using comparative analysis to the larger fungal genome collection was able to highlight pathogen-associated proteins highly conserved across fungal pathogens using the *F*-measure, as well as to recover all the proteins that are known effectors and to predict novel effector candidates due to cluster membership. Pathogen-associated proteins with high *F*-measure, i.e. proteins that score highly for pathogen or cereal-infecting specificity and conservation, are worthy candidates for putative virulence or pathogenicity factors common to a large set of pathogens. On the other hand, the SIX effectors and SnTox1 and SnTox3 have the lowest possible *F*-measure due to being exclusive to *F. oxysporum* and *S. nodorum*, respectively. However, for both genomes we find that these known effectors sit in clusters of proteins with strong signals for being small and secreted. This observation fits with known signatures for effectors and other proteins clustered with these known effectors based on similar sequence features may represent candidates worthy of further investigation. Similarly, other clusters of proteins that contain known effectors but are defined by different sets of sequence-derived features potentially represent uncharacterised effector properties. In summary, the *F*-measure and clustering methods are complementary approaches to analyze the space of pathogen-associated proteins. Fungal pathogens acting on the same host plant can be expected to utilize a common set of invasion strategies reflected in proteins highly conserved across fungal pathogens. At the same time, there will be effector proteins which are exclusively found in a particular fungal pathogen and as shown in this section, these can be found in an unbiased way using a clustering approach and subsequent protein selection step.

### Application to *F. graminearum*: pathogen-associated proteins highly conserved across fungal pathogens

After validation of the analysis pipeline on two relatively well characterized fungal pathogens (*S. nodorum* and *F. oxysporum*) with known effectors, the pipeline was applied to investigate the set of pathogen-associated proteins in one of the most important pathogens of cereals globally, the hemibiotrophic ascomycete *F. graminearum*[43]. As set in our pipeline, pathogen-associated proteins are returned initially and these are defined by having hits with more than 80% pathogen precision. Therefore, pathogen-associated proteins are proteins which are predominantly absent from the genomes of non-pathogens.

For *F. graminearum* proteins we find 2830 pathogen-associated proteins which represents 21% of all *F. graminearum* gene models (Additional file 3). We find that the vast majority (99%) of the 2830 *F. graminearum* proteins belong to the unclassified category when using MIPS Functional Catalogue Database (FunCatDB) for searching the functional distribution [44]. Only 241 of the 2830 proteins have a protein domain hit when sequence-based tools such as Pfam search are used. 3D structure similarities for 487 proteins of the 2830 proteins are reported by Phyre2 with confidence > 95% [45] and interestingly, 298 of these predicted protein structures have no Pfam domain hit. Thus, predicted 3D structure similarities can in many cases complement sequence-similarity based functional annotations.

The majority of the 2830 pathogen-associated proteins from *F. graminearum* (90%) have no significant sequence similarity hits to a fungal pathogen outside the *Fusarium* genus. The remaining 10% of the 2830 pathogen-associated proteins have sequence similarity hits to a diverse range of fungal pathogens. The 2830 pathogen-associated proteins from *F. graminearum* were ranked according to their degree of conservation across other fungal pathogens, with and without a cereal host, to reveal proteins highly relevant to pathogenicity and virulence. Table 1 shows the top 10 proteins in terms of cereal *F*-measure and pathogen *F*-measure, respectively, with their corresponding Pfam domain hits and predicted 3D structure similarities. We also report the taxonomical distribution of sequence similarity hits outside the MycoCosm tree by using an analysis of the phmmer web search with the non-redundant protein databank (NR) [32].

**Table 1:** **The top 10 pathogen-associated *F. graminearum *** **proteins in terms of cereal and pathogen F-measure**

**Protein id**	***F*-measure**	**Pfam domain (E-value, ID)**	**Phyre2 structure (Confidence/Coverage)**	**Molecular weight (kDA)**	**Signal peptide**	**Distribution of significant NR phmmer hits**
	**Cereal**					
FGSG_03333	0.71	Haloacid dehalogenase-like hydrolase (8e-10, PF12710)	Phosphorylcholine phosphatase (100%/90%)	41.1	Yes	93% Bacteria, 7% Ascomycota
FGSG_03338	0.7	–	Serine protease inhibitor 1 (98.4%/52%)	14.4	No	100% Ascomycota
FGSG_09148	0.69	Peptidase C69 (3.1e-25, PF03577)	Acyl-coenzyme (99.2%/44%)	57.2	Yes	90% Bacteria, 9% Eukaryota, 1% Archaea
FGSG_03339	0.66	–	Serine protease inhibitor 1 (99.2%/55%)	14.7	No	100% Ascomycota
FGSG_09328	0.66	Fungal specific transcription factor domain (3.5e-05, PF04082)	Centromere DNA-binding protein complex cbf3 (98.2%/72%)	62.6	No	94% Ascomycota, 6% Basidiomycota
FGSG_04015	0.66	–	–	57.4	No	100% Ascomycota
FGSG_03861	0.64	DUF3425 (1.8e-14, PF11095)	Pyrimidine pathway regulator 1 (99.4%/15%)	62.9	No	94% Ascomycota, 5% Basidiomycota, 1% others
FGSG_04507	0.63	C2 domain (1.1e-10, PF00168)	Endocytosis, exocytosis, synaptotagmin-1 (100%/54%)	52.1	No	41% Metazoan, 29% Viridiplantae, 22% Ascomycota, 8% others
FGSG_07909	0.62	Homeobox KN domain (2.3e-15, PF05920)	Homeobox domain (99.7%/11%)	84.6	Yes	56% Metazoan, 29% Viridiplantae, 12% Ascomycota, 3% others
FGSG_07846	0.61	FMO-like (5.8e-16, PF00734)	Monooxygenase (100%/75%)	62.6	No	37% Bacteria, 32% Metazoan, 15% Viridiplantae, 12% Ascomycota, 4% others
	**Pathogen**					
FGSG_04060	0.65	Rare lipoprotein A like double-psi beta barrel (3.1e-05, PF03330)	Beta-expansin 1a (100%/94%)	22.2	Yes	48% Bacteria, 16% Ascomycota, 13% Basidiomycota, 8% Phytophthora, 5% dictyostelium, 10% others
FGSG_09841	0.65	–	–	20.8	Yes	76% Ascomycota, 15% Bacteria, 6% Archaea, 3% others
FGSG_11496	0.64	Rare lipoprotein A like double-psi beta barrel (3.1e-05, PF03330)	Beta-expansin 1a (100%/93%)	25.2	Yes	23% Viridiplantae, 21% Bacteria, 18% Ascomycota, 13% Basidiomycota, 11% Phytophthora, 6% Dictyostelium, 8% others
FGSG_03333	0.58	Haloacid dehalogenase-like hydrolase (8e-10, PF12710)	Phosphorylcholine phosphatase (100%/90%)	41.1	Yes	93% Bacteria, 7% Ascomycota
FGSG_09148	0.55	Peptidase C69 (3.1e-25, PF03577)	Acyl-coenzyme (99.2%/44%)	57.2	Yes	90% Bacteria, 9% Eukaryota, 1% Archaea
FGSG_04507	0.52	C2 domain (1.1e-10, PF00168)	Endocytosis, exocytosis, synaptotagmin-1 (100%/54%)	52.1	No	41% Metazoan, 29% Viridiplantae, 22% Ascomycota, 8% others
FGSG_03549	0.52	–	–	28.2	No	100% Ascomycota
FGSG_11152	0.49	–	Coronatine-insensitive protein 1 (99.9%/94%)	44.4	No	100% Ascomycota
FGSG_09328	0.48	Fungal specific transcription factor domain (3.5e-05, PF04082)	Centromere DNA-binding protein complex cbf3 (98.2%/72%)	62.6	No	94% Ascomycota, 6% Basidiomycota
FGSG_07909	0.48	Homeobox KN domain (2.3e-15, PF05920)	Homeobox domain (99.7%/11%)	84.6	Yes	56% Metazoan, 29% Viridiplantae, 12% Ascomycota, 3% others

For each protein, its Pfam annotation, Phyre2 structure prediction, molecular weight and signal peptide predicted by SignalP are given as well as the distribution of significant phmmer hits against the non-redundant protein databank (NR) in terms of taxonomy.

**Figure 4:**
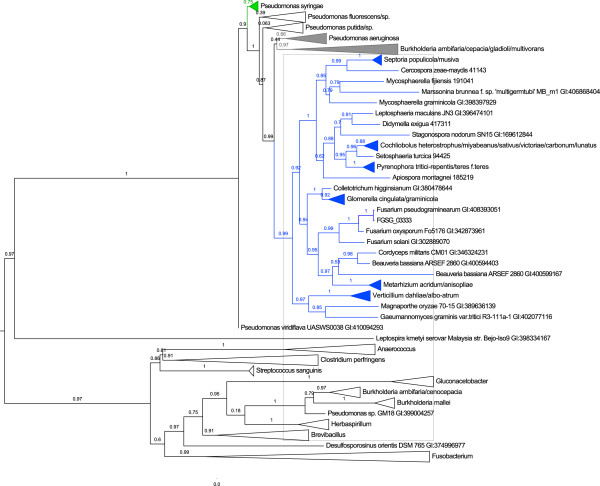
***FGSG_03333 *encodes a putative phosphorylcholine phosphatase with a shared selection process with bacterial plant pathogens.** For protein FGSG_03333, a BLASTp search returns 231 hits with E-value < 1.0e-05 and these were merged with our results from the MycoCosm tree. A MUSCLE alignment [48] and maximum likelihood phylogenetic tree estimation by PhyML [49] returns this phylogenetic tree with branch support values, visualized by FigTree [50]. The tree indicates that this protein has been selectively retained in hemibiotrophic and necrotrophic fungal pathogens of cereals, plants and insects. Furthermore, this protein is closely related to a highly conserved protein in a large number of Pseudomonads, indicating a shared selection process and suggesting a common role in pathogenicity.

A protein with a haloacid dehalogenase (HAD)-like hydrolase domain hit (FGSG_03333) has the highest cereal *F*-measure in our analysis. Using the Phyre2 structure prediction program FGSG_03333 is predicted to encode a phosphorylcholine phosphatase which is a subfamily of the HAD superfamily. Phosphorylcholine phosphatases have been associated with breakdown of the host cell membrane in bacteria [46]. FGSG_03333 is also predicted to be secreted and is orthologous to SNOG_009549 and FOXG_09820 identified in the analysis of the *S. nodorum* and *F. oxysporum* genomes (see above). A NCBI BLAST search and phylogenetic tree analysis reveals strong sequence-based matches to bacteria many of which are pathogenic on plants, e.g. *Pseudomonas syringae* (Figure 4). The I-TASSER server [47] also returns a confident 3D structure prediction (TM-score 0.61 ± 0.14) with the top structural analogue again a phosphorylcholine phosphatase from *P. aeruginosa*[46]. This *P. aeruginosa* protein has not previously been reported to be associated with cereal pathogenicity, however the phylogenetic analysis for FGSG_03333 suggests that its presence is characteristic of hemibiotrophic and necrotrophic cereal pathogens and that it may have undergone an ancient selection process with bacterial plant pathogens. Interestingly, we also find two proteins in close genomic proximity (FGSG_03338 and FGSG_03339) in the top 10 cereal F-measure table, which both have hits restricted to the Ascomycota (Table 1).

The highest pathogen *F*-measure of 0.65 is shared by two proteins and both are predicted to be secreted (FGSG_04060 and FGSG_09841). Protein FGSG_04060 is confidently predicted to be a beta-expansin 1a-like protein by Phyre2 as well as by the I-TASSER server (TM-score 0.73 ± 0.11), with the top structural analogue being a beta-expansin and group-1 pollen allergen from maize [51]. A public database search with FGSG_04060 returns a large number of significant hits to a wide range of pathogenic fungi including ascomycetes and basidiomycetes as well as to oomycetes and proteobacteria pathogenic on plants (Table 1). Bacterial and fungal expansin-like proteins have been associated with plant pathogenesis or plant cell wall degradation [52,53] and thus this protein might constitute a virulence factor that is worth functionally characterizing. Protein FGSG_09841 has an equally high pathogen *F-*measure with hits mainly to ascomycetes, however it has no significant Pfam domain hits or confident 3D structure predictions.

In summary, the ranking of pathogen-associated proteins in terms of their *F-*measure is a technique that is able to identify proteins in the query genome which are highly conserved across other fungal pathogens and at the same time predominantly absent from non-pathogenic fungi. Proteins with these properties are prime candidates for key factors in pathogenicity or virulence. The use of two different types of rankings, namely the cereal and pathogen *F-*measure aims to identify proteins that might be specific to pathogens acting on cereal hosts or on plants in general. All of the pathogen-associated proteins reported by us are predominantly absent from saprophyte genomes, however they vary in terms of how their sequence similarity hits are distributed in other kingdoms. For example, we find a large number of hits for FGSG_03333 to the bacterial kingdom or more specifically to bacterial plant pathogens, supporting a role in pathogenicity or virulence. On the other hand, for several of the proteins in Table 1 the taxonomical distribution outside the MycoCosm tree might not give a clear indication as to whether they have a functional role in virulence. For example, some proteins have sequence similarity hits restricted to pathogenic ascomycetes (FGSG_03338, FGSG_03339, FGSG_04015, FGSG_03549, FGSG_11152) whereas others contain domains that are widespread across the bacterial and eukaryotic kingdoms (FGSG_04507, FGSG_07909, FGSG_07846, FGSG_04060, FGSG_11496). Proteins are generally composed of several domains and the combination of the domains determines the function of the protein. Therefore, the presence of one domain that is widespread across pathogenic and non-pathogenic organisms, e.g. the F-box domain, and thus results in a large number of hits to diverse species, is not enough evidence to dismiss a protein as not having a role in pathogenicity or virulence.

### Clustering of the pathogen-associated proteins in *F. graminearum* reveals groups of putative effectors

To find putative *F. graminearum* effectors in the set of pathogen-associated proteins, we applied unsupervised clustering based on 35 sequence-derived features. As is required by the *k*-means approach, the number of clusters has to be predefined and it was set at 12, based on estimates using the elbow plot method (see Methods). The feature attributes of each cluster were compared to the full background distribution to identify those features that statistically discriminated that cluster from background. Clusters were examined further based on likely or established models of effector characteristics, for example a strong predicted secretion signal, potential novel signalling domain or a striking amino acid composition.

Table 2 shows the 12 clusters (numbered from C1 to C12) and their associated properties that were found using our *k-*means clustering approach (Additional file 4). We find three clusters of proteins (C2, C11, C12) with an enriched positive protein charge that are distinct from each other in terms of their amino acid composition. Cluster C12 contains proteins with a high percentage of aromatic amino acids, whereas cluster C2 features tiny amino acids and cluster C11 has a large proportion of polar amino acids. On the other hand, five clusters show depletion in positive protein charge and thus contain proteins with low charge (C3, C5, C8, C9, C10). Two of these clusters (C3, C9) contain a significantly higher number of aliphatic amino acids that are very hydrophobic and tend to stay in the interior of a protein structure. Cluster C5 shows depletion in cysteines and cluster C10 is enriched in aromatic amino acids. Another cluster (C6) contains 166 proteins with significantly higher molecular weight than the background distribution for all clusters, however no other discriminative features are evident. Cluster C4 has 210 proteins with enrichment of the amino acid proline. Proline has unusual biochemical properties and has been associated with a variety of functions, such as membrane proteins, cell adhesion or cereal storage proteins. Three clusters (C1, C7, C8) show an enriched secretion signal predicted by SignalP and WoLF PSORT, and are thus likely candidates for proteins which are secreted from the pathogen to the host apoplast.

**Table 2:** Selected properties for the clusters of pathogen-associated proteins

**Cluster**	**C1**	**C7**	**C8**	**C6**	**C3**	**C9**	**C5**	**C10**	**C11**	**C2**	**C4**	**C12**
**# Proteins**	**268**	**150**	**110**	**166**	**411**	**149**	**180**	**279**	**236**	**409**	**210**	**262**
**Characteristic**	**Secretion signal**			**Molecular weight**	**Hydrophobic**		**Negative charge**		**Positive charge**		**Proline**	**Aromatic**
**Secretion**	↑	↑	↑									
**Molecular weight**			↑	↑								
**Protein charge**			↓		↓	↓	↓	↓	↑	↑		↑
**Tiny**		↑	↑		↓		↓	↓		↑	↑	↓
**Small**		↑	↑		↓	↑		↓			↑	↓
**Aliphatic**	↑				↑	↑		↓	↓		↓	
**Aromatic**	↑		↓			↓	↓	↑	↓			↑
**Polar**	↓	↓			↓		↑		↑		↑	
**Charged**	↓	↓	↓				↑	↑	↑			
**Basic**	↓	↓	↓			↓			↑	↑		
**Acidic**	↓						↑			↓		
**Serine (S)**					↓					↑		
**Threonine (T)**			↑									
**Leucine (L)**	↑		↓		↑				↓			
**Cysteine (C)**		↑					↓					
**Glycine (G)**		↑										
**Proline (P)**											↑	

For each feature in the 35-dimensional feature vector, Mann–Whitney U tests were used to test whether the distribution within a cluster is identical to the full background distribution for all clusters and highly significant p-values for both directions (lesser ↓ and greater ↑, p-value < 2.2e-16) are shown. Secretion refers to the predicted SignalP score and WoLF PSORT extracellular score. The following amino acid membership are used: tiny (A,C,G,S,T), small (A,C,D,G,N,P,S,T,V), aliphatic (A,I,L,V), aromatic (F,H,W,Y), polar (D,E,H,K,N,Q,R,S,T), charged (D,E,H,K,R), basic (H, K, R) and acidic (D, E).

In general, we find that our *k*-means clustering approach divides the space of pathogen-associated proteins into groups of proteins with distinct sequence-derived features. As expected, some clusters have more discriminative features than others. We anticipate the set of pathogen-associated proteins to contain protein groups which share a number of features that are clearly distinct from background, as for example seen for small, secreted, cysteine-rich proteins in cluster C7. On the other hand, there will be protein groups which have been assigned to a cluster based on a less discriminative feature set, such as cluster C6 which contains proteins with high molecular weight and no other characteristic feature. Taken together, *k*-means clustering is an unsupervised method which will find natural groups of proteins with discriminative features in the set of pathogen-associated proteins. Unlike methods for effector prediction which use a number of thresholds and assumptions for selecting putative effectors, *k*-means clustering will return putative effector clusters in an unbiased way, if discriminative characteristics for effector proteins exist.

### Examination of putative effectors reveals extracellular domains and conserved N-terminal sequence motifs

From the clustering of pathogen-associated proteins and the extraction of significant cluster features, we identified three clusters (C1, C7, C8) of proteins with an enriched predicted secretion signal, thus prime candidates for containing putative effector proteins (Table 2). These three clusters differ significantly in terms of their protein characteristics and amino acid composition. Cluster C1 contains 268 proteins with enrichment in the secretion signal as well as in the non-polar, aromatic and aliphatic amino acid content. Aliphatic amino acids have been associated with transmembrane regions and indeed the TMHMM program [54] predicts transmembrane regions for 98 of the 268 proteins. Only 9 of the 268 proteins have a significant Pfam domain hit, e.g. a copper amine oxidase (FGSG_01761), a carbon-nitrogen hydrolase (FGSG_07204), an ion-channel (FGSG_11239) and a ferroportin 1 (FGSG_13446). The two other protein clusters (C7, C8) are enriched in tiny and small amino acids and are at the same time predicted to be secreted, which are features traditionally associated with effector proteins.

Cluster C7 contains 150 proteins and is enriched in amino acids cysteine and glycine. Cysteines are commonly associated with extracellular proteins due to their ability to form stable disulfide bonds. Glycine is the smallest amino acid and has no side chain. Thus glycine often plays a structural role in sterically restrictive turn regions. Significant Pfam domain hits are found for only 14 of the 150 proteins and some have associations with pathogenicity in the literature. FGSG_03573 has a predicted CFEM domain which has been found in extracellular fungal membrane proteins and has been associated with fungal pathogenesis, for example in *Magnaporthe grisea*[55]. Another protein FGSG_03109 has a cysteine-rich secretory Pfam domain hit and has predicted structural similarity to the plant PR-1 class of pathogen-related proteins. FGSG_00029 has a LysM domain which has been associated with carbohydrate and chitin binding and has been shown to be part of effector proteins in *Mycosphaerella graminicola*[56] and *Cladosporium fulvum*[57]. Phyre2 returns confident structure predictions for 31 of the 150 proteins, e.g. for putative beta expansions 1a (FGSG_04060, FGSG_11496) or a putative cellulose 1,4-beta-cellobiosidase fold (FGSG_07728). MEME does not find a motif common to more than five proteins from this set, except for the signal peptide. For example, FGSG_02841, FGSG_07766 and FGSG_08175 share common motifs as well as the three proteins FGSG_00111, FGSG_00112 and FGSG_13443. In summary, predicted extracellular protein domains and the enrichment in cysteines, small amino acids and the secretion signal form strong evidence for the presence of potential effector proteins in this cluster.

**Figure 5:**
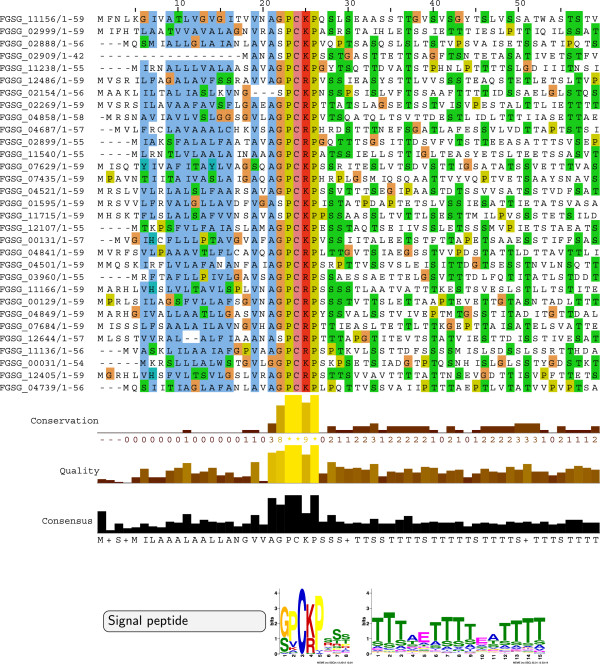
**A highly conserved N-terminal [SG]-P-C-[KR]-P motif in a subset of *F. graminearum *proteins.** MEME returns a [SG]-P-C-[KR]-P motif (e-value 3.4e-091) adjacent to the predicted signal peptide cleavage site for 37 of the 110 proteins in cluster C8. A MUSCLE alignment of the N-terminal region of a core set of well-aligned 31 of those proteins is visualized by Jalview. The MEME logos are shown with the [SG]-P-C-[KR]-P motif followed by a serine-/threonine-rich stretch. An iterative public database search of these aligned sequences (jackhmmer) returned additional members of this putative protein family in *F. graminearum*, *F. pseudograminearum, F. oxysporum* and *F. solani*.

Cluster C8 contains 110 proteins and has sequence-derived features that are distinct from Cluster C7. A striking difference to the other clusters is the enriched threonine content and molecular weight as well as the depleted leucine content and protein charge (Table 1). Interestingly, enrichment in serines, threonines and depletion in leucine has been reported for type III secreted effectors of animal and plant pathogens [3]. Significant Pfam domain hits are found for only 6 of the 110 proteins, i. e. for a WSC domain involved in carbohydrate binding (FGSG_09755, FGSG_10335, FGSG_11313, FGSG_11507), a pectate lyase domain (FGSG_13834) and a CFEM domain (FGSG_02109). Phyre2 returns confident structure predictions for 22 of the 110 proteins, e.g. a putative cellulose 1,4-beta-cellobiosidase fold (FGSG_02999) or a putative pectin lyase-like fold (FGSG_13834). For 37 of the proteins in this cluster, MEME returns a [SG]-P-C-[KR]-P motif (e-value 3.4e-091) adjacent to the predicted signal peptide cleavage site followed by a clear stretch of serines/threonines (Figure 5). We ran an iterative HMM search (jackhmmer) on the NR database with a MUSCLE alignment of the first 50 amino acids of a core set of well-aligned 31 sequences to look for the prevalence of this motif. After five iterations, we find 88 significant hits of the motif to proteins in only *F. graminearum*, *F. pseudograminearum*, *F. oxysporum* and *F. solani*. Subsets of these proteins share a common domain structure, however with no functional annotation for the majority of hits. A “G-P-C-R-P” motif was initially reported for *F. graminearum* in the Supplementary Material of Ma *et al.*[36], however it has not been functionally characterized. Zhang *et al.*[58] refer to this particular *F. graminearum* protein group as “CPGRP-anchored” (*sic*) and report transient induction at 16 h after inoculation during wheat coleoptile infection and at 2 to 8 h during *in vitro* germination. We suggest that this intriguing *Fusarium*-specific motif should be referred to as the [SG]-P-C-[KR]-P motif and that functional testing is necessary to reveal its role in the host-pathogen interaction.

**Figure 6:**
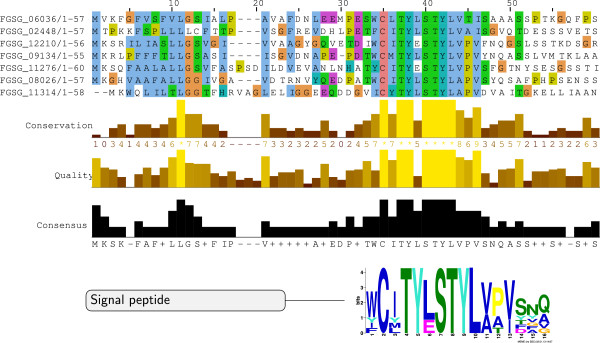
**A protein family with a conserved N-terminal [WYF]-C-x-T-Y-x-S-T-Y-L motif in *F. graminearum*.** MEME returns a [WYF]-C-x-T-Y-x-S-T-Y-L motif (e-value 1.2e-028) following the predicted signal peptide cleavage site for seven of the proteins in cluster C8. A MUSCLE alignment of the truncated 7 sequences in cluster C8 is visualized by Jalview.

For seven of the proteins lacking the [SG]-P-C-[KR]-P motif in Cluster C8, MEME predicts an alternative motif [WYF]-C-x-T-Y-x-S-T-Y-L about ten amino acids after the predicted N-terminal signal peptide (Figure 6). These 7 proteins are around 600 aas in length, have low average charge, and lack a functional annotation and confident 3D structure prediction. An iterative HMM search (jackhmmer) on the NR database with a MUSCLE alignment of the first 60 amino acids converged after three iterations. We collected 20 hits which are restricted to *F. graminearum*, *F. pseudograminearum, F. oxysporum*, *F. solani* and the cereal pathogen *Colletotrichum graminicola*. MEME predicts a highly conserved domain structure in the N-terminal region for these proteins (Additional file 5).

In summary, we have identified three protein clusters with an enrichment in the signal peptide prediction score for *F. graminearum*. These three clusters have distinct molecular features. Cluster C1 contains a high number of aliphatic amino acids and transmembrane proteins. The presence of extracellular domains and enriched cysteine content in cluster C7 indicates the occurrence of potential effectors in the set of 150 proteins. Cluster C8 has enriched threonine content, depleted leucine content and features proteins with high molecular weight and low charge. For proteins in this cluster, we predict two distinct conserved N-terminal motifs ([SG]-P-C-[KR]-P and [WYF]-C-x-T-Y-x-S-T-Y-L) which will be our prime candidates for functional characterisation in future work.

### *F. graminearum* putative effector clusters sit in regions of genome innovation

**Figure 7:**
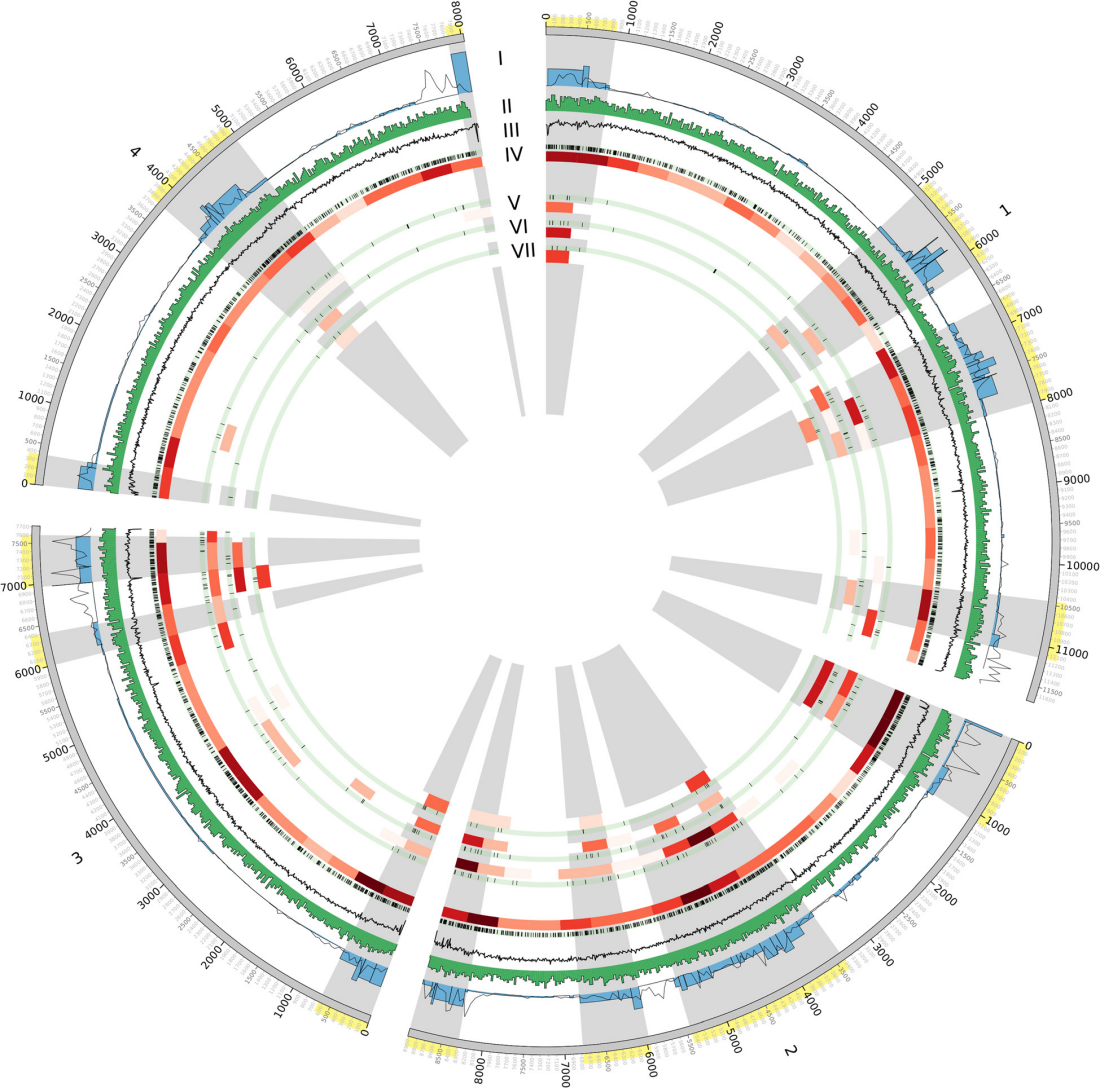
**Distribution of predicted protein clusters across the *F. graminearum *genome.** A Circos plot [62] is shown which visualizes the four *F. graminearum* chromosomes with ticks in kilobase (Kbp) units. The following bands for *F. graminearum* are visualized: **(I)** Recombination frequency (blue bars) and SNP density (line), **(II)** gene density, **(III)** GC content, **(IV)** set of 2830 pathogen-associated proteins, **(V)** protein cluster C7, **(VI)** protein cluster C8, **(VII)** proteins with the [SG]-P-C-[KR]-P motif. For each gene set, the location of the genes on the chromosomes and a heatmap in red shading for the gene count in 500 Kbp bins are shown. It can be seen that proteins from putative effector clusters C7 and C8 occur predominantly in regions of genome innovation.

Subtelomeric and ancient sub-telomeric regions of the *F. graminearum* genome have been previously described as regions of genome innovation and are characterised by higher levels of genetic diversity between strains and other *Fusarium* compared to the rest of the genome, higher density of secreted and *in planta* expressed proteins and higher genetic recombination rates per unit length of DNA [43,59]. Pathogenicity genes from the Pathogen-Host Interactions database (PHI-base [60]) have been negatively associated with these regions [61]. However, these pathogenicity genes are dominated by highly conserved components of fungal biology such as signalling cascades which when mutated render the fungus with many pleiotropic phenotypes. Genes associated with virulence may have different distributions across the genome compared to pathogenicity genes. Analysis of the clusters of genes identified in this work reveals distinct distributions across the *F. graminearum* chromosomes (Figure 7). Members of cluster C1, which is enriched in secretion signals and features a number of proteins with transmembrane regions, are found throughout the genome (data not shown). In contrast, members of putative effector clusters C7 and C8 and especially proteins containing the [SG]-P-C-[KR]-P motif are found in regions of genome innovation as depicted in Figure 7.

### De novo motif search reveals potential signalling, targeting or uptake motifs in *F. graminearum*

The aims of our study were to find the set of pathogen-associated proteins in *F. graminearum* and to dissect them in terms of their pathogenicity and virulence potential as well as their effector potential in an unbiased way. In this section, we applied *de novo* motif search to look for novel effector sequence signatures in the set of pathogen-associated proteins. Effector proteins are active outside the fungal cell and can be expected to carry a secretion signal. Bioinformatics signal peptide prediction programs such as SignalP [63] use neural networks and HMMs for predictions and thus rely on the quality of the training data. Especially for fungal pathogens, the data on known secretion signals or host-translocation motifs is scarce and thus, we applied *de novo* motif search to look for novel sequence signatures. We expect sequence signatures comprising potential secretion signals and host-translocation motifs to occur within the 150 N-terminal residues of a protein. We apply the motif search tool MEME to each predicted cluster and report motifs which occur on average within the first 150 residues of the proteins and are present in more than five proteins.

To validate our approach for finding conserved N-terminal sequence signatures with potential function in signalling, targeting or uptake, we applied our pipeline to the powdery mildew fungus *Blumeria graminis* f. sp. *hordei* which infects barley [64]. An N-terminal Y/F/WxC-motif has been described in powdery mildew fungal effector candidates [65]. We applied MEME to the pathogen-associated protein clusters predicted by our pipeline in *B. graminis* f. sp. *hordei* (Additional file 6). We find that only one of the clusters has a significantly higher secretion signal than the other clusters and for 98 of the proteins in this cluster, MEME predicts an N-terminal Y/F/WxC-motif (Additional files 7 and 8). Our set of 98 proteins predicted from the *B. graminis f. sp. hordei* is of similar size to the set of 107 proteins identified by Godfrey *et al.*[65] from a set of around 3000 genes from sequenced ESTs. After positive validation of our motif search approach in the space of pathogen-associated protein clusters in *B. graminis f. sp. hordei*, we applied it to *F. graminearum*.

For *F. graminearum*, the *de novo* sequence motif prediction results for the protein N-terminal regions within the 12 clusters are shown in Table 3. For each of the motifs found in the clusters, we built a HMM from the multiple alignment computed by MUSCLE and used an iterative, sensitive jackhmmer search against the non-redundant protein databank (NR), with a maximum of five rounds, to record the prevalence of the motifs. As reported in the previous section, we find two motifs in cluster 8 which are largely restricted to pathogenic *Fusarium* and occur, on average, within the first 50 residues of the proteins and after a predicted signal peptide. Another motif sits in cluster C12 and shares resemblance to the L-P-x-E motif which occurs in F-box like domains. An iterative jackhammer search finds that this motif is exclusive to Ascomycota with hits predominantly to fungal pathogens, for example most prominently to *S. nodorum* or *Pyrenophora tritici-repentis*. In future work we will experimentally test these motifs for their functional role in the host-pathogen interaction.

**Table 3:** ***De novo *** **N-terminal motif search results for the pathogen-associated protein clusters in F. graminearum**

	**Motif logo and regular expression**	**Prevalence in cluster**	**E-value**	**Average start position in protein**	**Comments**
Cluster C4		10 sites	3.5e-31	143	Domain seems widespread throughout eukaryotic kingdom.
	Q[QS]QQQQQ[QH]QQ[QDM][QAP][QP]Q[QH]QQ[QM][QN]Q[QS]QQ[QL][QH]Q[QAP]QQ[QA][QAP][QM][QL][QP]Q[QP][QH][QP][QL][QH]Q				
		13 sites	8.0e-24	56	Domain seems restricted to fungal kingdom, large number of hits predominantly to the Ascomycota. Some protein hits are annotated as fungal transcriptional regulatory proteins or GTP binding protein.
	AC[DE]RC[RK]R[LR]K[IT][KR]C[DS]				
		6 sites	5.1e-03	48	Domain seems restricted to fungal kingdom, large number of hits predominantly to the Ascomycota. Some protein hits are annotated with fungal transcriptional regulatory function. Weak Pfam domain annotation as zinc cluster.
	CSRCV[KR]xKLDC[DE]Y				
Cluster C5		6 sites	9.2e-03	4	Domain seems widespread throughout eukaryotic kingdom. There are weak similarities to zinc-finger protein domain.
	MCVxV[HT]KDITC[PS]TC				
Cluster C8		37 sites	9.3e-91	15	Motif is adjacent to predicted signal peptide cleavage site. Domain is restricted to pathogenic *Fusarium* genomes in our search.
	[AL][LA]Axx[AV]xA[GS]PC[KR]PSS				
		8 sites	1.6e-29	47	Search converged after three iterations. Domain is restricted to *F. graminearum*, *F. oxysporum*, *F. solani* and the cereal pathogen *G. graminicola*.
	WCITY[LE]STYL[VA][PA][VI]SN				
Cluster C12		11 sites	2.4e-16	11	Domain restricted to fungal kingdom, hits are predominantly to Ascomycota including a large number of pathogens, e.g. *S. nodorum*. Weak hit to PRANC domain, which is found at the C-terminus of Pox virus proteins. L-P-x-E motif is also found in F-box like domains.
	FH[PL]F[SL]RLPPE[LIV]RL[MQ]I[WY]RHALT				

We report motifs in *F. graminearum* which are distinct from convential signal peptides and which are conserved in more than 5 sequences and occur within the first 150 amino acids to identify potential novel signalling, subcellular targetting or uptake motifs.

## Conclusions

The bioinformatics search for fungal pathogen proteins involved in attack on the host plant has thus far focussed on finding small and cysteine-rich peptides in the predicted fungal secretome [20]. Therefore, such methods have been limited in their ability to return putative effectors with those previously recognized features. However, there is growing evidence in the literature that effectors can have unconventional characteristics, such as no predicted signal peptide, a low number of cysteine residues or a large size [21-23,25]. Here, we take advantage of the availability of sequenced fungal genomes and present a novel and unbiased method for isolating genes strongly associated with pathogenic species using comparative genomics analysis followed by an unsupervised clustering step using multiple protein properties. This type of approach allows us to predict putative pathogen-associated proteins or effectors with previously unrecognized characteristics. The main contributions of our method are as follows.

### Beyond BLAST: protein comparisons in fast-evolving genome regions

For finding proteins in a query pathogen genome which are conserved across other pathogens and predominantly absent from non-pathogens, we use a HMM methodology [32] as a sensitive method for finding pathogen-associated proteins. Sensitive HMM methods are particularly well-suited for comparative analysis in fast-evolving genome regions, such as pathogenicity islands or dispensable chromosomes. Unlike previous studies which made use of the stringent reciprocal best BLAST hit method [28,29], our pipeline is able to detect remote sequence similarity relationships across a large number of genomes and protein clusters sharing domains and common structural features derived from their peptide sequences. The reciprocal best BLAST hit method and the triangular extension of clusters of orthologous groups (COGs) are efficient tools for deriving orthology across genomic sequences, however they are inadequate for large evolutionary distances as found in fast-evolving genome regions [66]. Orthology is a powerful concept, however ideally phylogenetic methods should be used in fast-evolving pathogen genomes instead of reciprocal best BLAST hits and COGs and for these, a resolved fungal tree of life would be a prerequisite.

### *F*-measure ranking for finding virulence mechanisms common and exclusive to pathogens

We introduce a novel way for ranking pathogen-associated proteins in a query genome according to their degree of conservation across other fungal pathogens, with and without a cereal host, using the *F*-measure. This allows for the identification of fungal pathogen proteins that are widely distributed across other pathogenic fungi and at the same time predominantly absent from non-pathogenic fungi. Using this methodology, we were able to identify a highly ranked putative secreted phosphorylcholine phosphatase in *F. graminearum* for which a phylogenetic analysis suggests that it is characteristic of necrotrophic and hemibiotrophic pathogens and has undergone a shared selection process with bacterial plant pathogens. Proteins which are highly ranked according to our *F*-measure method are prime candidates for proteins essential for a pathogenic lifestyle and virulence.

### Clustering approach returns known effectors and novel effector candidates

We use a *k*-means unsupervised clustering approach to divide the space of pathogen-associated proteins into natural groups with shared sequence-derived features. This is a step towards finding structural similarities within the pathogen-associated proteins in the twilight zone of sequence similarity. By using an unsupervised clustering approach on the set of pathogenic proteins without the use of heuristic thresholds, we can predict putative effectors which would have gone undetected with pipeline approaches that rely on heuristic assumptions. We demonstrate that our pipeline returns known effectors in *S. nodorum* and *F. oxysporum.* For *F. graminearum*, we predict three clusters with a high secretion signal that contain prime candidates for effector proteins. We found that the proteins of two of these putative effector clusters are found predominantly in regions of genome innovation. One of the clusters is enriched in threonines and molecular weight as well as depleted in leucines and protein charge. Enrichment of threonines and depletion of leucines is a characteristic of type III secreted proteins in animals and plants [3]. Within this cluster, we identified two putative protein families that each have a distinct conserved sequence motif adjacent to the predicted signal peptide cleavage site. One of them is a highly conserved N-terminal [SG]-P-C-[KR]-P motif exclusive to *Fusarium* with unknown function. We found several other N-terminal motifs conserved in proteins lacking a predicted secretion signal, e.g. a L-P-x-E-R motif with remote homology to F-box domains that is highly conserved in pathogenic Ascomycota such as *S. nodorum*. In future work we will experimentally test these motifs for their functional role, i.e. whether they might be signalling, subcellular targetting or uptake motifs in the host-pathogen interaction.

### Quality of genomic data and application to other genomic data sets

Our bioinformatics pipeline can be applied to any pathogenic genome for which a contrasting set of non-pathogenic relatives is available, such as publicly available fungal or bacterial genomes. It should be emphasized that accuracy and sensitivity of any comparative genomics analysis relies heavily on the quality of genome assembly and subsequent gene prediction and annotation. Databases housing a large number of genome projects such as the JGI MycoCosm will inevitably feature entries of diverse annotation quality. Gene boundary prediction errors are of particular concern for effector candidates which are expected to harbour N-terminal motifs such as signal peptides. However, unlike fungal effector prediction pipelines that strictly select effector candidates based on a predicted signal peptide, our clustering approach can to some extent compensate for incorrect gene boundaries. For example, the *F. oxysporum* SIX2 effector for which the software SIGNALP 4.0 does not predict a signal peptide is still placed in the same cluster as the secreted SIX1, SIX6, SIX9 and SIX10 effectors based on its sequence-derived features. This might indicate that our clustering approach successfully recognizes structural similarities amongst the pathogen-associated proteins and can offset sporadic gene boundary prediction errors. However, improvements in gene assembly tools and automated gene prediction pipelines are of utmost importance and our method will naturally be able to take advantage of the increasing number of fungal genomes available and of improved genome annotation quality.

Before taking promising effector candidates to experimental validation, one can incorporate expression data of *in planta* infection such as microarray or RNAseq data. The pipeline scripts are available from the authors on request. With regard to the cereal pathogen genome set, already pre-processed datasets are available for further analysis.

## Methods

### Fungal genomes and proteomes

Predicted protein sets from 172 fungal genomes were downloaded from the DOE Joint Genome Institute MycoCosm web site [33] and supplemented with the *B. graminis* f. sp. *hordei* genome [64] and our in-house genome assembly for *F. pseudograminearum* CS3096 (GenBank: AFNW00000000.1, [29]). The majority of MycoCosm genomes are of non-pathogenic fungi. More specifically, 56 of the 174 fungal genomes belong to pathogenic fungi and 19 are fungal pathogens of a cereal host (Table 4).

**Table 4:** The set of 19 fungal pathogen genomes used in our comparative study which have a cereal host

**Genus**	**Fungal genome**	**Cereal host range**	**Lifestyle**	**Reference**
*Fusarium*	*Fusarium graminearum*	Wheat, barley, maize	Hemibiotroph	[43]
	*Fusarium pseudograminearum*	Wheat, barley, maize	Hemibiotroph	[29]
*Magnaporthe*	*Magnaporthe grisea*	Rice, barley	Hemibiotroph	[67]
*Puccinia*	*Puccinia graminis* f. sp. *tritici*	Wheat	Biotroph	[17]
*Ustilago*	*Ustilago maydis*	Maize	Biotroph	[68]
*Cercospora*	*Cercospora zeae-maydis*	Maize	Necrotroph	-
*Cochliobolus*	*Cochliobolus carbonum 26-R-13*	Maize	Necrotroph	-
	*Cochliobolus heterostrophus C4*	Maize	Necrotroph	[69]
	*Cochliobolus heterostrophus C5*	Maize	Necrotroph	[69]
	*Cochliobolus lunatus m118*	Sorghum	Necrotroph	-
	*Cochliobolus miyabeanus ATCC 44560*	Rice	Necrotroph	-
	*Cochliobolus sativus ND90Pr*	Cereal generalist	Hemibiotroph	[69]
	*Cochliobolus victoriae FI3*	Oat	Necrotroph	-
*Mycosphaerella*	*Mycosphaerella graminicola*	Wheat	Hemibiotroph	[70]
*Pyrenophora*	*Pyrenophora teres f. teres*	Barley	Necrotroph	[71]
	*Pyrenophora tritici-repentis*	Wheat	Necrotroph	[69]
*Setosphaeria*	*Setosphaeria turcica Et28A*	Maize	Hemibiotroph	[69]
*Stagonospora*	*Stagonospora nodorum SN15*	Wheat	Necrotroph	[35]
*Blumeria*	*Blumeria graminis* f.sp. *hordei*	Barley	Biotroph	[64]

{TC “ 1 The set of 19 fungal pathogen genomes from the MycoCosm tree which have a cereal hots.”\f t}.

### One-against-all proteome HMM comparisons

Sensitive proteome comparisons are used to identify proteins in the query genome of interest which are conserved across other fungal genomes and share a similar protein domain structure. One of the pathogenic fungal genomes is set as the query genome of interest. For the protein set of the query genome, the set of significant protein hits across the 174 fungal genomes is calculated using the profile HMM search tool phmmer [32]. This returns a list of hits with full-sequence E-values for each protein from the query genome. Proteins are composed of one or more domains. There can be multiple matches across a query, i.e. protein domain hits, and these are returned by phmmer as a table of per-domain hits. A query protein is said to have a significant match in another fungal genome if there is a full-sequence hit with E-value < 10^-5^ and if the combined domain hits cover more than 60% of the query and target sequences, respectively. For each query protein, its significant fungal genome matches are recorded.

### Comparative analysis: pathogen-associated proteins

As a result of the phmmer searches, each protein in the query genome has a number of protein hits across the list of 174 fungal genomes, including to itself. To decide if a query protein *x*_*i*_ is specific to fungal pathogens and highly conserved across fungal pathogens, four measurements of the relevance of the phylogenetic distribution of the phmmer hits with respect to pathogenicity are calculated: cereal-recall *R*^*c*^ (*i*), pathogen-recall *R*^*p*^ (*i*), cereal-precision *P*^*c*^ (*i*) and pathogen-precision *P*^*p*^ (*i*). These values are defined as follows:

(1) $$R^{c}\left(i\right)=100\times \frac{\mathrm{hits}\;\mathrm{to}\;\mathrm{cereal}\;\mathrm{pathogens}}{\mathrm{cereal}\;\mathrm{pathogens}},$$

(2) $$R^{p}\left(i\right)=100\times \frac{\mathrm{hits}\;\mathrm{to}\;\mathrm{fungal}\;\mathrm{pathogens}}{\mathrm{fungal}\;\mathrm{pathogens}},$$

(3) $$P^{c}\left(i\right)=100\times \frac{\mathrm{hits}\;\mathrm{to}\;\mathrm{cereal}\;\mathrm{pathogens}}{\mathrm{hits}},$$

(4) $$P^{p}\left(i\right)=100\times \frac{\mathrm{hits}\;\mathrm{to}\;\mathrm{fungal}\;\mathrm{pathogens}}{\mathrm{hits}}.$$

A high recall means that a method returns the majority of relevant results. High precision means that a method returns more relevant results than irrelevant results. If a protein has high cereal- or pathogen-recall it means that its phmmer hits cover the majority of fungal pathogens with or without a cereal host, respectively. If a protein has high cereal- or pathogen-precision it means that its phmmer hits mostly contain fungal pathogens with or without a cereal host, respectively. Precision and recall can be combined into one measurement, the *F*-measure:

(5) $$F=2\times \frac{\mathrm{precision}\times \mathrm{recall}}{\mathrm{precision}+\mathrm{recall}}.$$

For each query protein *x*_*i*_, recall and precision for its hits across the 174 fungal genomes are recorded. Proteins *x*_*i*_ are kept with pathogen-precision of *P*^*p*^ (*i*) > 80%, i.e. at least 80% of its phmmer hits are to other pathogenic fungi, and we call these pathogen-associated proteins. These pathogen-associated proteins are ranked according to their *F*-measure in terms of cereal and fungal pathogen hits.

### Clustering of pathogen-associated proteins

Centroid-based clustering with the *k*-means method was used to find clusters of similar query proteins in the set of pathogen-associated proteins *S*. Each protein *x*_*i*_ ∈ *S* has a 35-dimensional feature vector based on its sequence information. Let the feature vector *v*_*i*_ for protein be given as

$$v_{i}=\langle \left|v_{i}\right|,s_{i},e_{i},m_{i},h_{i},l_{i},p_{i}^{1},\ldots ,p_{i}^{9},a_{i}^{1},\ldots ,a_{i}^{20}\rangle ,$$

where |*v*_*i*_| is the protein length. Let *s*_*i*_ be the *D*-score returned by SIGNALP 4.0, which corresponds to the prediction whether the sequence contains a signal peptide or not [63]. Let *e*_*i*_ be the score for extracellular localization site prediction calculated by WoLF PSORT [72]. The molecular weight of a protein is given by *m*_*i*_, the charge of a protein is given by *h*_*i*_ and the isoelectric point is given by *l*_*i*_. Furthermore, the chemical properties of the amino acids in the protein are used as a first step to detect structural homologues with little sequence identity [73]. Let $p_{i}^{1},\ldots ,p_{i}^{9}$ be the percentage of amino acids in a protein which are classified as tiny, small, aliphatic, aromatic, non-polar, polar, charged, basic and acidic, respectively. These features are calculated using pepstats from the EMBOSS package [74]. Let $a_{i}^{1},\ldots ,a_{i}^{20}$ be the percentage of amino acid residues in a protein. The entries in the 35-dimensional feature vectors are numerical and were normalized to avoid a bias towards a feature with higher range. Centroid-based clustering with the *k*-means method requires the number of clusters to be specified in advance and we use the heuristic elbow plot method for this purpose. For each feature in the 35-dimensional feature vector, Mann–Whitney *U* tests were used to test whether the distribution within a cluster is identical to the full background distribution, i.e. all clusters, and highly significant p-values for both directions (lesser and greater, p-value < 2.2e-16) are recorded.

### Functional annotation and motif search

3D protein structure predictions were obtained using the remote homology recognition technique Phyre2 [45]. Phyre2 is able to predict 3D structure similarities in cases when sequence similarity search tools fail. For Phyre2, a confidence threshold of 95% was used. For selected proteins, the computationally more demanding I-TASSER server was used [47]. The TM-score returned by I-TASSER measures the structural similarity between two structures. A TM-score < 0.17 means that the result is of random similarity. A TM-score > 0.5 indicates that the model is of correct topology. The C-score returned by I-TASSER is a confidence score for estimating the quality of predicted models by and is typically in the range of from −5 to 2. A high C-score relates to a model with a high confidence. Protein domain searches were performed using a local Pfam search for each query protein against Pfam’s library of profile HMMs [32]. Hits with E-value < 10^-5^ are considered as significant hits. The tool MEME was used for sequence motif discovery [75]. MEME was run with a minimum and maximum motif width of 4 and 50, respectively, and set to return the best 30 motifs.

### Implementation of the comparative HMM analysis pipeline

All analyses described above were conducted with custom Python and R scripts. The *k*-means clustering was performed using SciPy and the Mann–Whitney *U* tests were carried out using R.

### Availability of supporting data

The following additional data are available with the online version of this paper. Additional file 1 is a table listing the properties of the predicted pathogen-associated proteins in *S. nodorum*. Additional file 2 is a table listing the properties of the predicted pathogen-associated proteins in *F. oxysporum*. Additional file 3 is a table listing the properties of the predicted pathogen-associated proteins in *F. graminearum* as well as the individual clusters and predicted motifs. Additional file 4 contains box plots for the 12 clusters in *F. graminearum* and the 35 protein features used for the clustering. Additional file 5 contains the full sequence alignment for the proteins containing the motif [WYF]-C-x-T-Y-x-S-T-Y-L. Additional file 6 is a table listing the properties of the predicted pathogen-associated proteins in *B. graminis*. Additional files 7 and 8 contain information about the predicted Y/F/WxC-motif proteins *B. graminis*.

## Competing interests

The authors declare that they have no competing interests.

## Authors’ contributions

JS developed and implemented the bioinformatics pipeline, performed data analysis and drafted the manuscript. All authors contributed to the design and development of the bioinformatics pipeline. DMG and JMM analysed the biological data and predictions. JMT performed statistical data analysis. JKH assisted in the development of the bioinformatics pipeline and the genomic analysis. JS, DMG, JMT, KBS and JMM conceived the study. All authors contributed to the draft and read and approved the final version.

## Supplementary Material

Additional file 1 **Table listing the properties of the predicted pathogen-associated proteins in *S. nodorum*.** Click here for file

Additional file 2 **Table listing the properties of the predicted pathogen-associated proteins in *F. oxysporum*.** Click here for file

Additional file 3 **Table listing the properties of the predicted pathogen-associated proteins in *F. graminearum *** **as well as the individual clusters and predicted motifs.** Click here for file

Additional file 4 **Box plots for the 12 clusters in *F. graminearum *** **and the 35 protein features used for the clustering.** Click here for file

Additional file 5 Contains the full sequence alignment for the proteins containing the motif [WYF]-C-x-T-Y-x-S-T-Y-L. Click here for file

Additional file 6 **Table listing the properties of the predicted pathogen-associated proteins in *B. graminis*.** Click here for file

Additional file 7 **Contain information about the predicted Y/F/WxC-motif proteins *B. graminis*.** Click here for file

Additional file 8 **Contain information about the predicted Y/F/WxC-motif proteins *B. graminis*.** Click here for file
