# Supplementary material for: A comparative hidden Markov model analysis pipeline identifies proteins characteristic of cereal-infecting fungi
Source: BMC Genomics. 2013 Nov 20;14:807. doi: 10.1186/1471-2164-14-807 (PMC3914424; doi:10.1186/1471-2164-14-807)
Supplement: Additional file 4 — Box plots for the 12 clusters in F. graminearum and the 35 protein features used for the clustering. [file 1471-2164-14-807-S4.pdf]

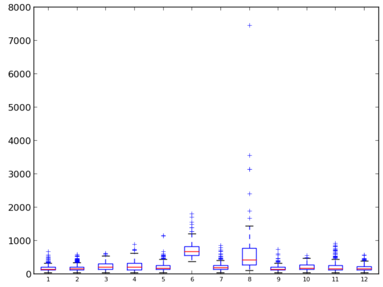

(a) Sequence length.

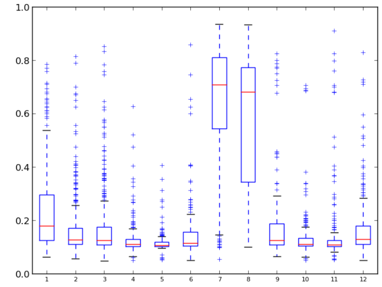

(b) SIGNALP 4.0 score.

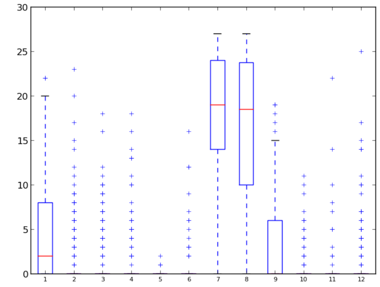

(c) WoLF PSORT extracellular score.

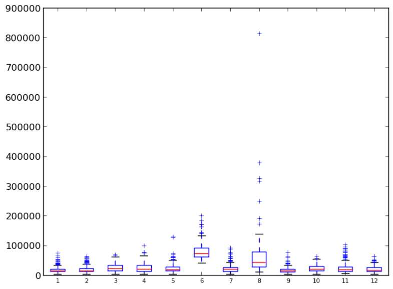

(d) Molecular weight.

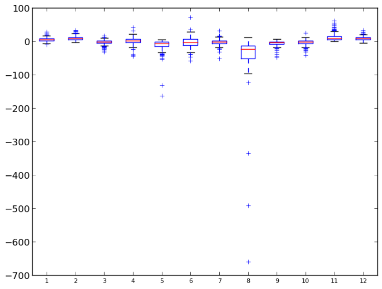

(e) Protein charge.

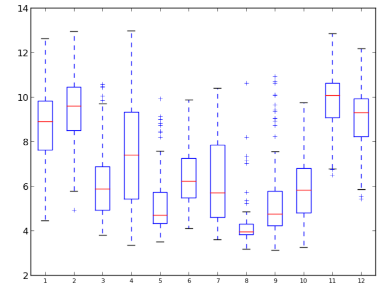

(f) Isoelectric point.

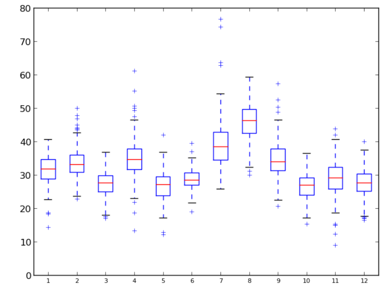

(g) Tiny amino acids.

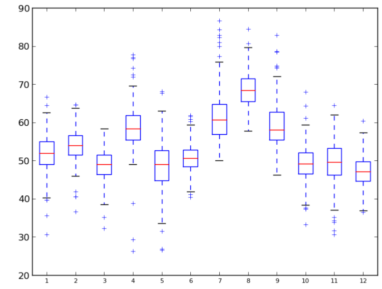

(h) Small amino acids.

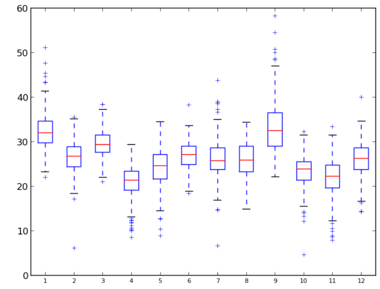

(i) Aliphatic amino acids.

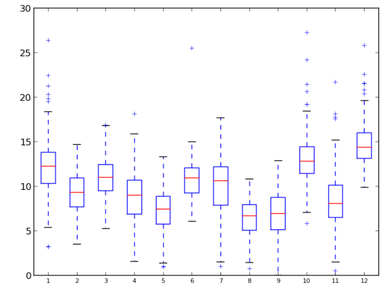

(j) Aromatic amino acids.

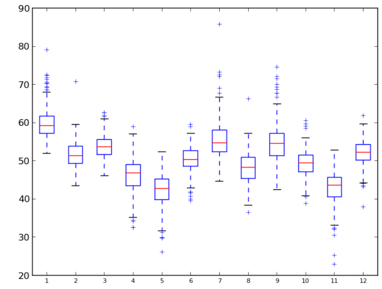

(k) Nonpolar amino acids.

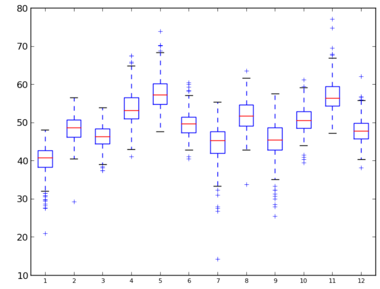

(l) Polar amino acids.

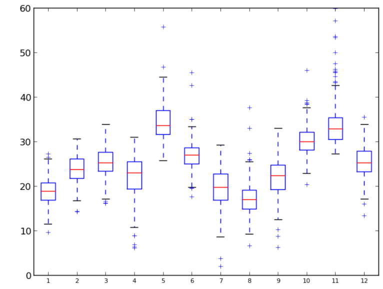

(m) Charged amino acids.

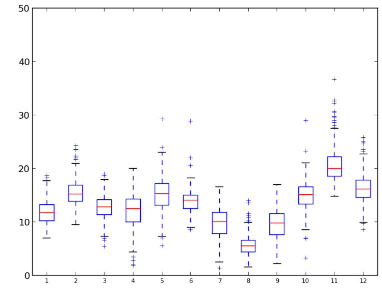

(n) Basic amino acids.

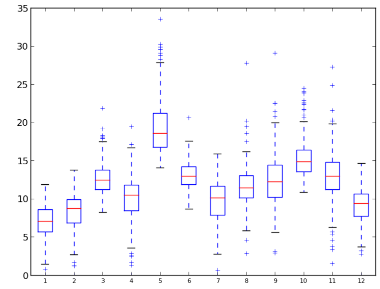

(o) Acidic amino acids.

Figure 1: *F.graminearum* box plots for the 12 clusters and the 35 protein features used for the clustering. The smallest observation, lower quartile, median, upper quartile, largest observation and outliers are shown.

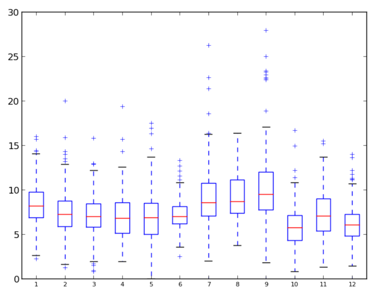

(a) Amino acid alanine (Ala, A).

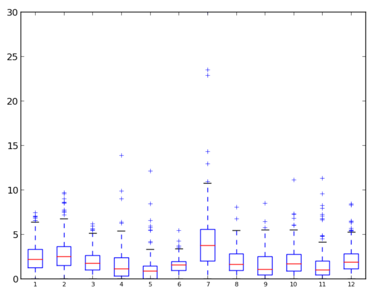

(b) Amino acid cysteine (Cys, C).

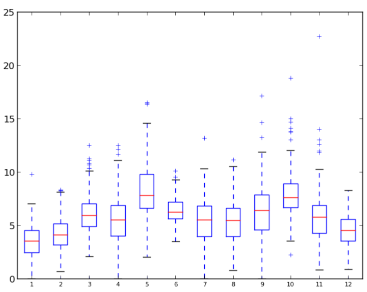

(c) Amino acid aspartic acid (Asp, D).

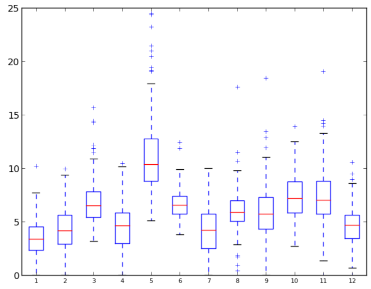

(d) Amino acid glutamic acid (Glu, E).

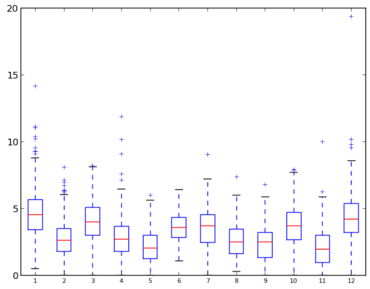

(e) Amino acid phenylalanine (Phe, F).

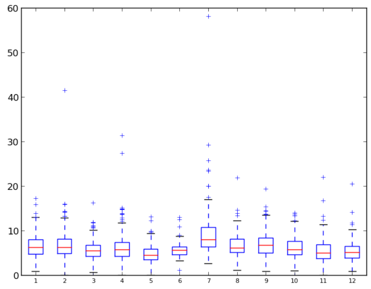

(f) Amino acid glycine (Gly, G).

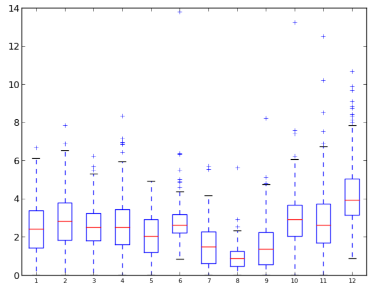

(g) Amino acid histidine (His, H).

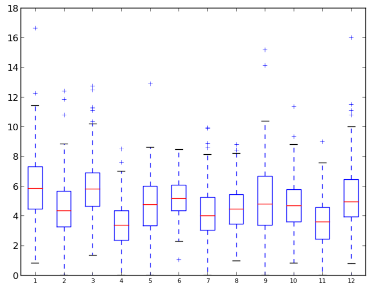

(h) Amino acid isoleucine (Ile, I).

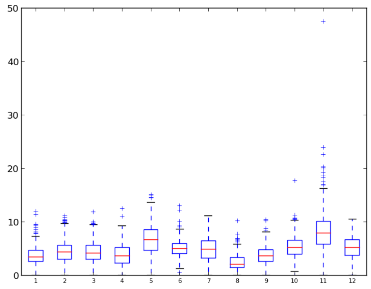

(i) Amino acid lysine (Lys, K).

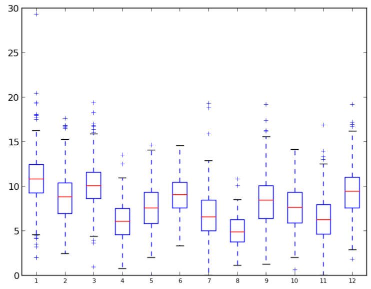

(j) Amino acid leucine (Leu, L).

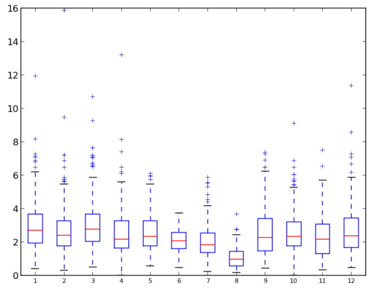

(k) Amino acid methionine (Met, M).

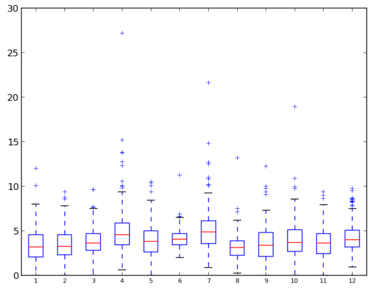

(l) Amino acid asparagine (Asn, N).

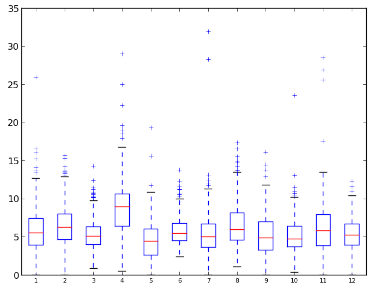

(m) Amino acid proline (Pro, P).

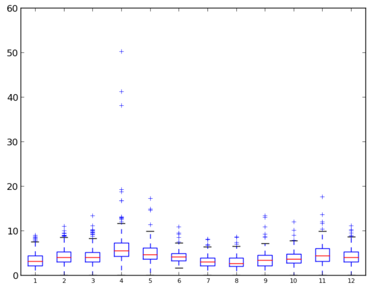

(n) Amino acid glutamine (Gln, Q).

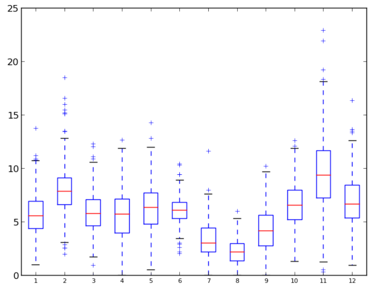

(o) Amino acid arginine (Arg, R).

Figure 2: *F.graminearum* box plots for the 12 clusters and the 35 protein features used for the clustering. The smallest observation, lower quartile, median, upper quartile, largest observation and outliers are shown.

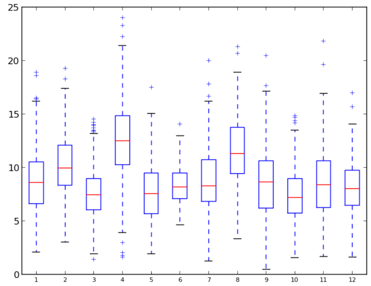

(a) Amino acid serine (Ser, S).

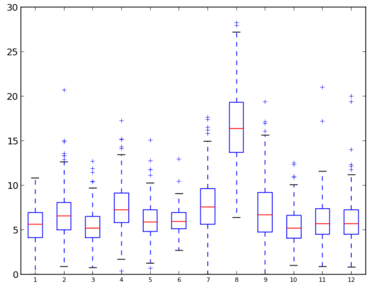

(b) Amino acid threonine (Thr, T).

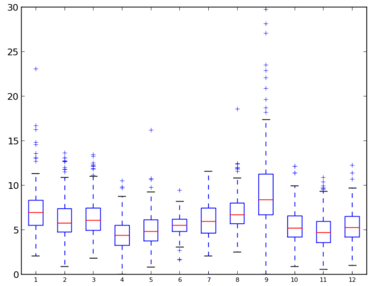

(c) Amino acid valine (Val, V).

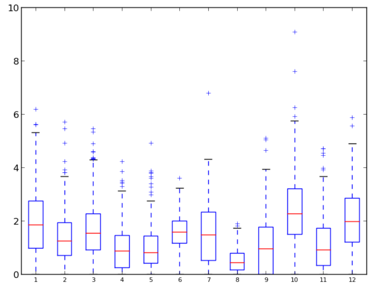

(d) Amino acid tryptophan (Trp, W).

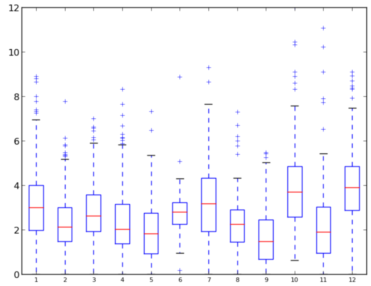

(e) Amino acid tyrosine (Tyr, Y).

Figure 3: *F.graminearum* box plots for the 12 clusters and the 35 protein features used for the clustering. The smallest observation, lower quartile, median, upper quartile, largest observation and outliers are shown.
