# Supplementary figures and images for: A comparative hidden Markov model analysis pipeline identifies proteins characteristic of cereal-infecting fungi
Source: BMC Genomics. 2013 Nov 20;14:807. doi: 10.1186/1471-2164-14-807 (PMC3914424; doi:10.1186/1471-2164-14-807)

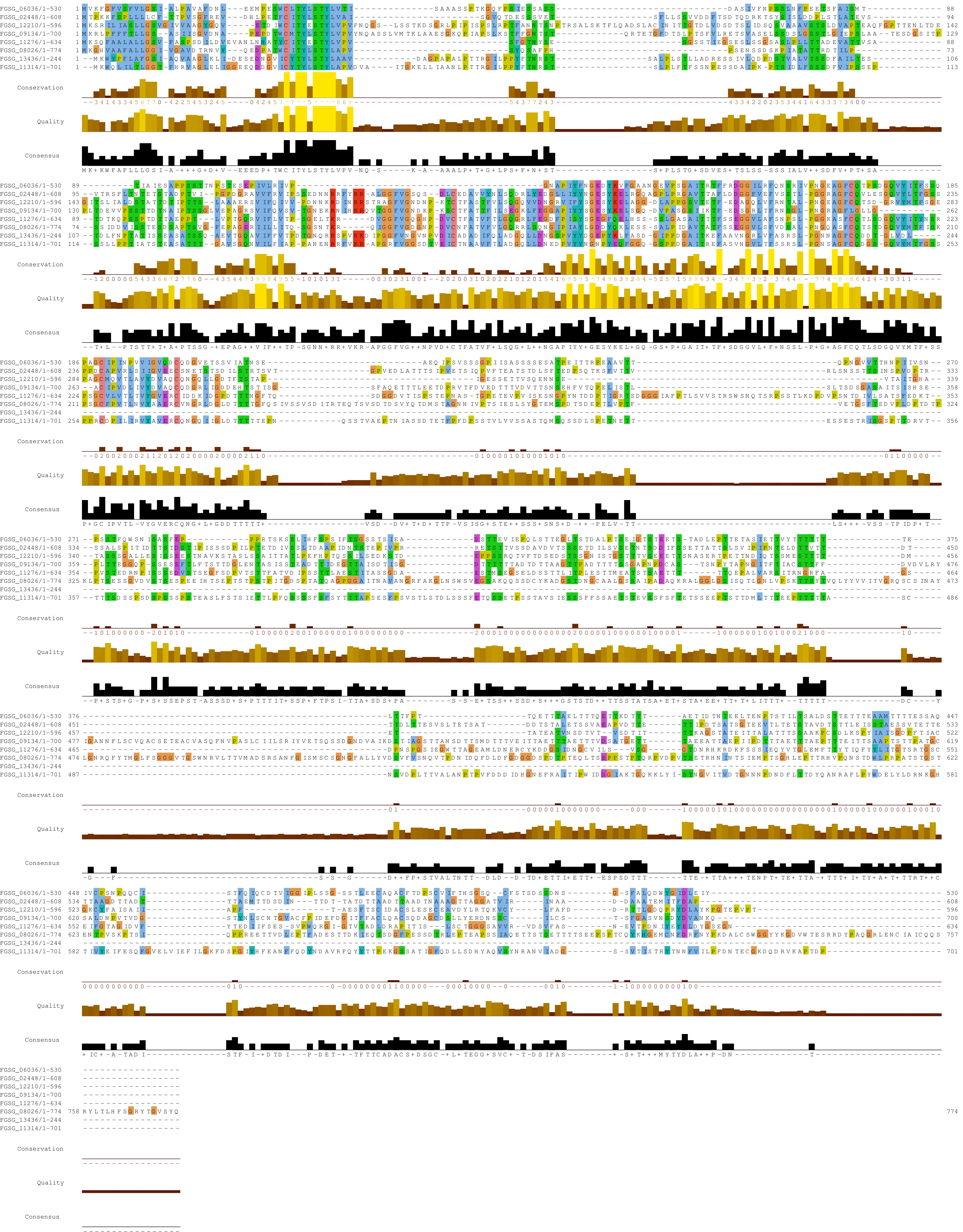

Supplement: Additional file 5 — Contains the full sequence alignment for the proteins containing the motif [WYF]-C-x-T-Y-x-S-T-Y-L. [file 1471-2164-14-807-S5.pdf]

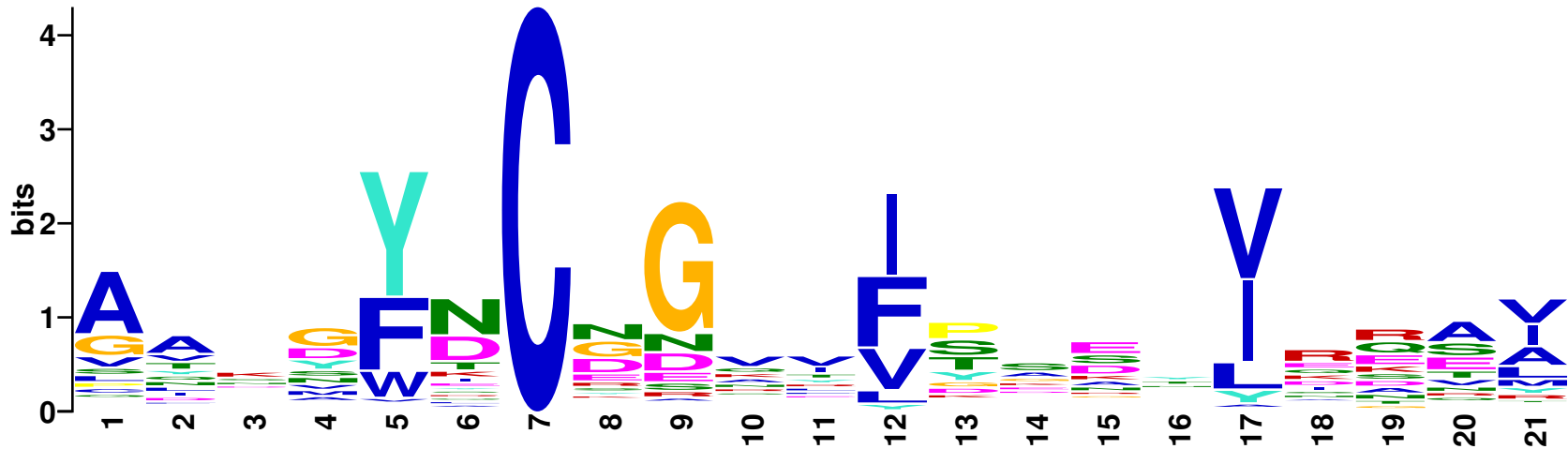

Supplement: Additional file 8 — Contain information about the predicted Y/F/WxC-motif proteins B. graminis. [file 1471-2164-14-807-S8.pdf]
